# Room Temperature Quantum Spin Hall Insulator in Ethynyl-Derivative Functionalized Stanene Films

**DOI:** 10.1038/srep18879

**Published:** 2016-01-05

**Authors:** Run-wu Zhang, Chang-wen Zhang, Wei-xiao Ji, Sheng-shi Li, Shi-shen Yan, Shu-jun Hu, Ping Li, Pei-ji Wang, Feng Li

**Affiliations:** 1 School of Physics and Technology, University of Jinan, Jinan, Shandong, 250022, People’s Republic of China; 2 School of Physics, State Key laboratory of Crystal Materials, Shandong University, Jinan, Shandong, 250100, People’s Republic of China

## Abstract

Quantum spin Hall (QSH) insulators feature edge states that topologically protected from backscattering. However, the major obstacles to application for QSH effect are the lack of suitable QSH insulators with a large bulk gap. Based on first-principles calculations, we predict a class of large-gap QSH insulators in ethynyl-derivative functionalized stanene (SnC_2_X; X = H, F, Cl, Br, I), allowing for viable applications at room temperature. Noticeably, the SnC_2_Cl, SnC_2_Br, and SnC_2_I are QSH insulators with a bulk gap of ~0.2 eV, while the SnC_2_H and SnC_2_F can be transformed into QSH insulator under the tensile strains. A single pair of topologically protected helical edge states is established for the edge of these systems with the Dirac point locating at the bulk gap, and their QSH states are confirmed with topological invariant Z_2_ = 1. The films on BN substrate also maintain a nontrivial large-gap QSH effect, which harbors a Dirac cone lying within the band gap. These findings may shed new light in future design and fabrication of large-gap QSH insulators based on two-dimensional honeycomb lattices in spintronics.

Topological insulators (TIs)^1^,^2^,^3^,^4^ have generated intensive research and engineering activities in recent years, because they give an alternative and robust platform for obtaining relativistic and spin-polarized Fermions in the condensed matter system. One of the most interesting phenomena in this realm is the quantum spin Hall (QSH) effect in two-dimensional (2D) structures^1^,^2^, characterized by an insulating bulk and gapless edge states at its boundaries due to time-reversal symmetry (TRS), thus providing enticing concepts for novel quantum devices with low energy dissipation^3^,^4^. The prototypical concept of QSH effect is first proposed by Kane and Mele in graphene^5^,^6^, in which the spin-orbit coupling (SOC) opens a band gap at the Dirac point. However, the associated gap due to rather weak SOC is too small (~10^–3^ meV), which makes the QSH state in graphene only appear at an unrealistically low temperature. To date, quantized conductance through QSH state has only been experimentally observed in HgTe/CdTe^7^,^8^ and InAs/GaSb^9^,^10^ quantum wells at the ultralow temperature, which greatly obstructs further experimental studies and possible applications in spintronics.

Generally, a good QSH insulator should be easily synthesized and have a large bulk band gap to realize the spin transport at high temperatures. Controlling the chemical bonding at atomic levels to induce the band inversion by orbital selection^3^,^4^ is an efficient way to realize QSH effect^8^,^11^,^12^,^13^,^14^,^15^. 2D materials are advantageous in this respect as their bonding character is relatively easy to modify in post synthesis processes, for example, by surface adsorption. Recently, group-IV honeycomb lattices such as functionalized germanene and stanene^16^,^17^ have been reported, with a bulk-gap as large as 0.2–0.3 eV, sufficiently large for practical applications at room temperature. Besides, the strong SOC effect can also be sufficed by group-V heavy elements such as bismuth, which drives nontrivial QSH states^18^,^19^. On the other hand, group III-V materials GaAs and GaBi have also reported to be large-gap QSH insulators^20^,^21^. Unfortunately, the experiments^22^ reveal that plasma fluorination and hydrogenation generally increase defects and lattice disorder even under short plasma exposures. Thus, the achievement of these systems with high quality is rather challengeable, demonstrating the detection of QSH effect is difficult in experiments. More recently, an approach to design a large-gap QSH state on a semiconductor surface by a substrate orbital filtering process is also proposed^23^,^24^. These large-gap QSH insulators are essential for realizing many exotic phenomena and for fabricating new quantum devices that can operate at room temperature.

Recently, the small molecule functionalization has been the focus to enhance the geometric stability and nontrivial band gap of new 2D films. For instance, Methyl (CH_3_), another organic molecule, has also been suggested as a promising tool to stabilize 2D systems, such as methyl-functionalized germanane (GeCH_3_)^25^ and bismuth (BiCH_3_)^26^, to realize large gap QSH insulators. Experimentally, GeCH_3_ film has been synthesized in recent work^27^. This raises an interesting question: can the methyl be applied to stabilize group IV and V films, and whether their band gap can be enhanced significantly in QSH phase? Motivated by recent works on stanene by zhang *et al.*^17^, in our previous works^28^, we have found that the organic molecule ethynyl (C_2_H) can be applied to stabilize stanene by decoration on its surface, and its band gap in TI states reaches up to 0.3 eV. However, the QSH phase is not intrinsic, but driven by the external strain, thus is unfavorable for practical applications in spintronics. Here, we extend these into ethynyl-derivative functionalized films (SnC_2_X; X = F, Cl, Br, I), making the experimental observation facile. The SnC_2_Cl, SnC_2_Br, and SnC_2_I are all QSH insulators with a bulk gap of ~0.2 eV, while the SnC_2_H and SnC_2_F can be transformed into QSH insulator under the tensile strains. The topological characteristic of these films is attributed to *s-p*_*xy*_ band inversion related to the honeycomb symmetry, while the effect of SOC is only to open up a large gap. We also propose high-dielectric BN as an ideal substrate for the experimental realization of these films, maintaining its nontrivial topology. These results have potential applications in low-power quantum electronics and may enable topological quantum computing based on Majorana fermions^29^.

## Results and Discussion

**Figure 1:**
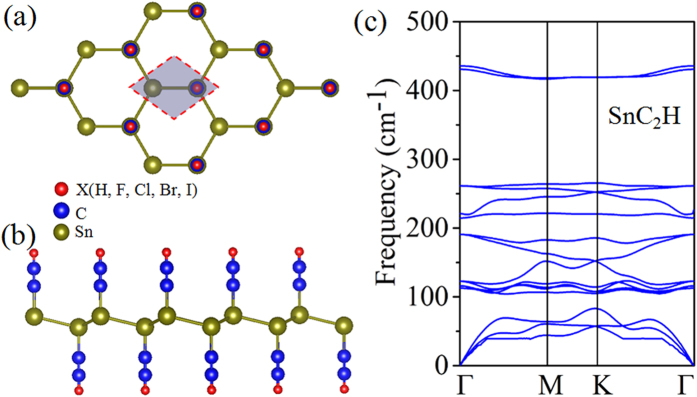
(**a**) Side and (**b**) top views of the atomic structures of SnC_2_X (X = H, F, Cl, Br, I). Red, blue, and green balls denote X, C, and Sn atoms, respectively. Shadow area in (**a**) present the unit cell. Phonon band dispersions of (**c**) denote SnC_2_H film.

We have performed first-principles calculations of electronic structures and band topology of 2D hexagonal lattices functionalized with ethynyl-derivatives. We first discuss in detail the results of SnC_2_H and SnC_2_Br as representative examples. Figure 1(a) shows the geometric structure of 2D SnC_2_X, which prefers a buckled configuration, with C_2_X bonding on both sides of stanene in an alternating way (Fig. 1(b)), in analogy to hydrogenated germanene and stanene^16^,^17^. In comparison to the pristine stanene, the lattice constant of SnC_2_X increase slightly by 0.19 ~ 0.06 Å, along with the buckling height between Sn planes decreasing by about 0.14 ~ 0.07 Å, the details as listed in Table 1. The buckling plays a crucial role in the engineering of the band structures in this structure. To confirm the structural stability, we calculate the phonon spectrum along the highly symmetric directions, as shown in Fig. 1(c). One can see that all branches of the phonon dispersion curves have positive frequencies and no imaginary phonon modes are found, confirming the stability of SnC_2_H film.

**Figure 2:**
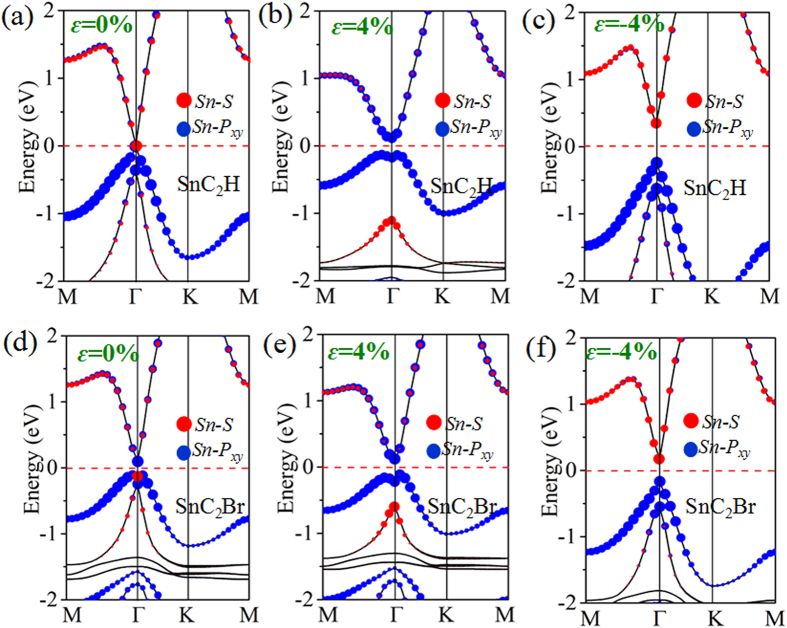
Orbital-resolved band structures with SOC of SnC_2_H (a–c) and SnC_2_Br (d–f) under biaxial strain *ε* = 0%, 4% and −4%, respectively. The red dots represent the contributions from the *s* atomic orbital of Sn atom and the blue dots represent contributions from the *p*_x_ and *p*_y_ atomic orbitals of Sn atom.

To get a preliminary insight into the topological properties, we present the calculated band structures of SnC_2_H including SOC. At equilibrium state in Fig. 2(a), which shows a semiconductor nature with a direct band gap located at Γ point. By projecting the bands onto different atomic orbitals, we find that the valence band maximum (VBM) at Γ point near the Fermi level is mainly composed of *p*_x,y_ orbitals from Sn atoms with the features of binding states, whereas the conduction band minimum (CBM) is the anti-binding *s* state. Thus, no inverted band order is observed, suggesting that it is a trivial TI phase. However, if applying the tensile strain (*ε* = 4%), the *s-p* band inversion appears, with an indirect-gap of 0.22 eV opened under SOC effect, as illustrated in Fig. 2(b). To demonstrate the 2D TI phase, we calculate the Z_2_ invariants *v*, as listed in Table 2. Obviously, the products of the parity eigenvalues at these three symmetry points: Γ (0.0, 0.0), M_1_ (0.5, 0.0) and M_2_ (0.0, 0.5) are both 1, while at the M_3_ (0.5, 0.5) shows −1, which give Z_2_ = 1. Therefore, it directly confirms the existence of QSH insulator state.

**Figure 3:**
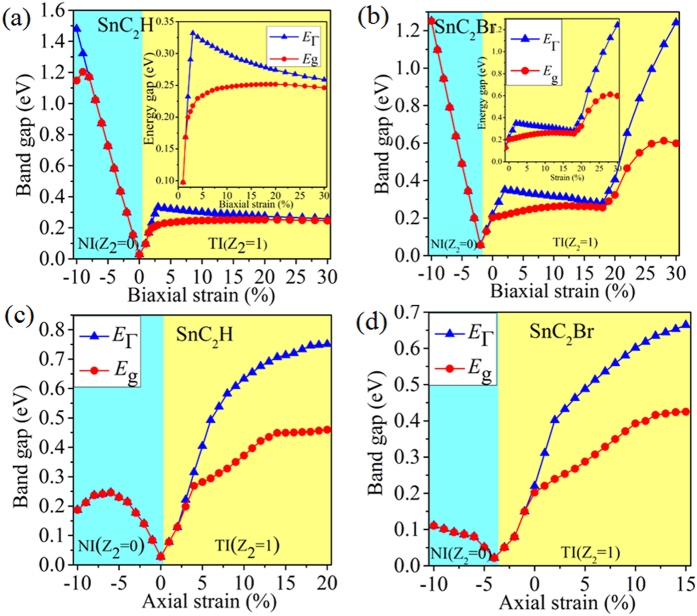
The calculated band gaps at Γ point (*E*_Γ_) and the global band gap (*E*_g_) of SnC_2_H and SnC_2_Br with SOC as a function of biaxail and uniaxial strains. Notably, the insets in panel show the trend of band gaps of TI phase as a function of external biaxial strain.

The buckled configuration generally sustain a larger mechanical strain than planner one, thus we explore the variations of electronic structure with respect to the mechanical strain^30^,^31^,^32^,^33^,^34^. Here, we employ an external strain on SnC_2_H maintaining the crystal symmetry by changing its lattices as *ε* = (a − a_0_)/a_0_, where a (a_0_) is the strained (equilibrium) lattice constants. In what follows, we systematically investigate the effects of both uniaxial and biaxial strains on topological properties of SnC_2_H. Figure 3(a) presents the variation of nontrivial QSH gap (direct, *E*_Γ_) at Γ point and bulk gap (indirect, *E*_g_) as a function of the biaxial strain. One can see that, both the direct and indirect band gaps in TI phase decrease steadily with respect to strain, especially *E*_g_ and *E*_Γ_ being equal to strain beyond 30%. Remarkably, for *ε* = 3%, we find the largest gap of ~0.33 eV opened at Γ point, along with a global indirect band gap of ~0.22 eV, which is significantly greater than *k*_*B*_*T* (~26 meV). While the biaxial strain is compressed to *ε* = −4%, we find that no inverted band order is observed, suggesting that it is a trivial QSH insulator (Fig. 2(c)). On the other hand, we explore the effect of uniaxial strain on the electronic properties, as displayed in Fig. 3(c). Interestingly, we obtain a larger SOC-induced bulk gap as large as ~0.46 eV (*ε* = 18%), which is almost twice the one of biaxial strain. Further analysis of the parity eigenvalue gives Z_2_ = 1, which suggests that the uniaxial strain is also efficient way to tune QSH state in these films.

Now, we wish to point out that the ethynyl functionalization in stanene is not the only way to achieve the large-gap QSH state, the same results can be obtained by decorating the surface with otherwise ethynyl-derivatives, such as -C_2_F, -C_2_Cl, -C_2_Br, and -C_2_I. We thus perform calculations for SnC_2_X (X = F, Cl, Br, I) films to check their topological properties. Table 1 summarizes their lattice constants, Sn-Sn bond lengths, and nontrivial gaps at their equilibrium states. Similar to the case of SnC_2_H, for SnC_2_F, it is a trivial QSH phase at the equilibrium state, but can be driven to QSH phase at the biaxial tensile strain *ε* > 2%. However, for the other SnC_2_X (X = Cl, Br, I) films, they are TI phases at the equilibrium state with Z_2_ = 1, as listed in Table 2. The global QSH gaps of SnC_2_Cl, SnC_2_Br, and SnC_2_I are 0.19, 0.20, and 0.23 eV, respectively, (see Table 1 and supplemental information in Fig. S1), which are sufficiently large for practical application at high temperature.

The calculated band structures of SnC_2_Br film are presented in Fig. 2(d–f) as a typical model. Obviously, the *s*-*p*_xy_ band inversion are still exist, in analogy to the strain-induced QSH phase in SnC_2_H. Similar results for SnC_2_F, SnC_2_Cl, and SnC_2_I films are also observed, as illustrated in Fig. S1. From Fig. 3(b), we also find that the biaxial-strain range of QSH phase is rather large (from −3% to 30%). Interestingly, there are two peaks in the region of the topology phase, in which one peak is 0.34 eV for *E*_g_ under 3% strain, and another higher peak is 0.61 eV at 28%. Such robust topology makes it easier for experimental realization in spintronics. For uniaxial strain (Fig. 3(c–d)), we consider the effects of uniaxial strain along x direction for SnC_2_H and SnC_2_Br. In addition, the detailed evolution of band structure for SnC_2_H under the uniaxial strain is added into Fig. S2. Band structure calculations indicate that it is still in QSH phase, with a larger bulk gap of ~0.42 eV (*ε* = 16%). Also, as can be seen in Fig. S3, the systematically investigate the effects of both uniaxial and biaxial strains on topological properties of SnC_2_F, SnC_2_Cl and SnC_2_I, respectively, which are sufficiently tunable ranges of strain for practical applications at high temperature.

**Figure 4:**
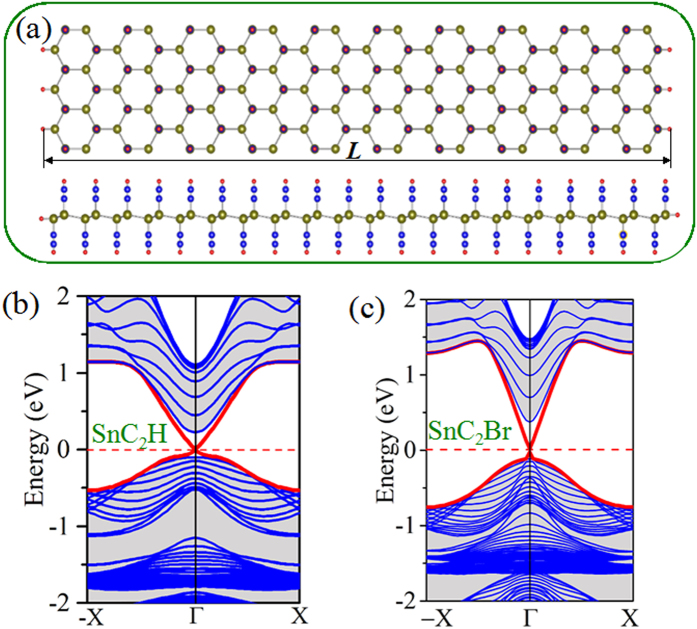
(**a**) Schematic structure of the zigzag-type nanoribbon of SnC_2_H and SnC_2_Br films. (**b**) and (**c**) indicate the band structures of zigzag-type edge states in QSH phase. The helical edge states are indicated by the red lines.

The QSH phase should support topologically protected conducting edge states that are helical with the spin-momentum locked by TRS. To demonstrate these edge states explicitly, we take SnC_2_H and SnC_2_Br with the external strain *ε* = 2% and *ε* = 0%, respectively, to introduce edges on stanene by forming the zigzag-type nanoribbons, as shown in Fig. 4(a). The edge Sn atoms are passivated by hydrogen atoms to eliminate the dangling bonds. The width of the nanoribbon is large enough to avoid interactions between the edge states of the two sides. Figure 4(b,c) show the zigzag-type edge states of SnC_2_X (X = H, Br) films, where a Dirac point at the *Γ* point is located inside the band gap with a high velocity of ~1.0 × 10^5^ m/s and ~2.0 × 10^5^ m/s, comparable to that of 5.5 × 10^5^ m/s in HgTe/CdTe quantum well^35^, both of which are larger than that of 3.0 × 10^4^ m/s in InAs/GaSb quantum well^36^. Helical edge states are very useful for electronics and spintronics owning to their robustness against scattering. The similar results for the remaining edge states of SnC_2_X (X = F, Cl, I) are also displayed in Fig. S4. All these band structures maintain topologically protected conducting edge states that are helical, thus providing enticing concepts for novel quantum electronic devices with low energy dissipation.

It is known that GGA method usually underestimates the band gap, we thus carried out test computations based on the hybrid functional HSE06^37^,^38^ to assess the robustness of our results within the PBE. The calculated band structures of SnC_2_X (X = Cl, Br, I) films under the critical strain are presented in Fig. S3 in supplementary information. One can see that the band gap of these films are indeed enhanced. However, the *s*-*p*_*x,y*_ band order is not changed and in agreement with those obtained from PBE, indicating the PBE results are qualitatively reasonable and reliable.

**Figure 5:**
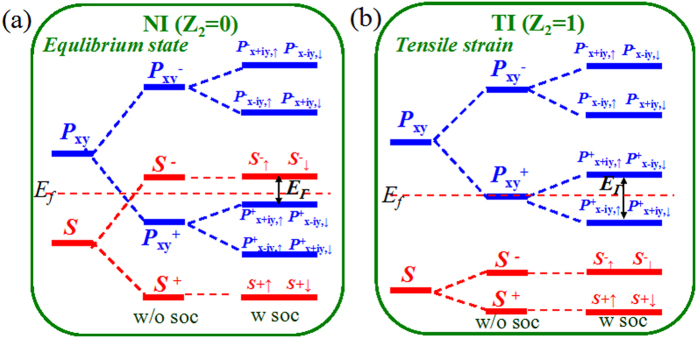
The evolution of atomic *s* and *p*_x,y_ orbitals into the band edges at Γ point of SnC_2_X (X = H, F) at (a) equilibrium state, and (b) tensile strain (ε = 2%). The horizontal red dashed lines in (**a**,**b**) indicate the Fermi level.

To better understand the physical origin of QSH states, we next do an orbital analysis around the Fermi level for SnC_2_X films. Figure 5 presents the band evolution at Γ point, in which the energy levels near the Fermi level are mainly composed of Sn-5*s* and Sn-5*p*_x,y_ orbitals. Thus, the chemical bonding between Sn-Sn atoms makes them split into the bonding and antibonding states, *i.e., s*^±^ and *p*_x,y_^±^, where the superscripts + and − represent the parities of corresponding states, respectively. At zero strain in Fig. 5(a), the bands near the Fermi level are contributed by the *p*_x,y_^+^ and *s*^−^, with the *s*^−^ being above the *p*_x,y_^+^, indicating a normal band order, as shown in SnC_2_H. When considering the tensile strain, as seen in Fig. 5(b), the enlarged lattice constant weakens the interaction between Sn atoms, decreasing the splitting between the bonding and antibonding states, which lowers *s*^−^ level and raises *p*_x,y_^+^ level accordingly. Thus, depending on the strength of interatomic coupling, the band gap of SnC_2_H can be continuously reduced, with the band order being reversed at critical point, making it a topological insulator with Z_2_ = 1. Taken together, the level crossing leads to a parity exchange between occupied and unoccupied bands, inducing a TI phase transition. While for SnC_2_Br, the lattice constant at equilibrium state is equal to strain-induced SnC_2_H (*ε* = 4%), thus it is a QSH insulator. As a result, the *s*-*p* band inversion^39^ is a strong indication of the existence of topologically nontrivial phases. Herein, we must point out that, although the *s*-*p*_x,y_ band inversion is caused mainly by the strength of interatomic coupling, the SOC is still indispensable because it makes the *p*_x,y_^+^ orbital split into *p*^+^_x + iy, ↑_ & *p*^+^_x−iy, ↓_ and *p*^+^_x−iy, ↑_ & *p*^+^_x+iy, ↓_, opening a larger band gap. In previous works, the topological states are usually originated from *p*_z_ system such as graphene and silicene of which the nontrivial gaps are often very small^40^. but in our cases, the topological states with larger gaps are obtained due to the contribution of *p*_x,y_ orbitals on Sn atoms, where the large SOC is from the on-site SOC, instead of the next-nearest-neighbor SOC in the *p*_z_ cases^40^. These results emphasize the importance of orbital selection for topological materials.

**Figure 6:**
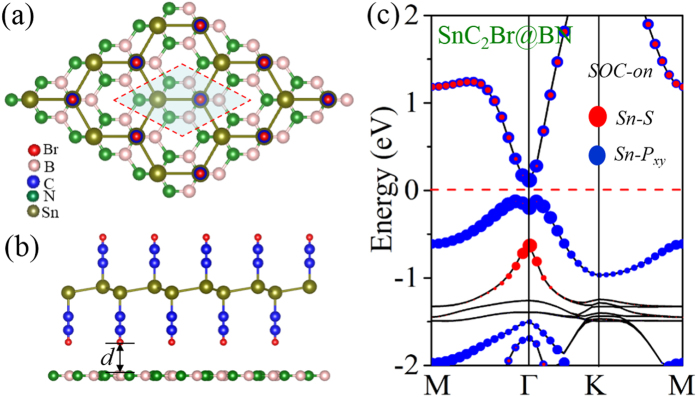
(**a**) Top and (**b**) side views of the schematic illustration of the epitaxial growth SnC_2_Br on *h*-BN (2 × 2) substrate. (**c**) is orbital-resolved band structure with SOC for SnC_2_Br@BN.

On the experimental side, choosing the suitable substrate material is a key factor in device application, since a free-standing film must eventually be deposited or grown on a substrate. It is known that the QSH features in pristine graphene and germanene^12^,^13^,^14^,^15^,^16^,^17^ are easily destroyed by the substrate. In contrast, although the TI phase of SnC_2_X films are obtained in free-standing structure, their nontrivial QSH would be robust when they are on the substrate, because their band inversion occurs at Γ point rather than at K point, as well as the full saturation of Sn-*p*_z_ orbitals ensures a weak interaction with the substrate. Here, we select *h*-BN as a possible substrate to form SnC_2_Br@BN heterostructure (HTS), where the lattice mismatch is only about 1.19% (SnC_2_Br) in comparison to *h*-BN(2 × 2) sheet, showing that they are feasible to grown on the *h*-BN substrate. To correctly describe the effect of van der Waals (vdW) interaction, we employ a dispersion-corrected DFT method (optB88-vdW)^41^,^42^, which has been demonstrated to reliably describe 2D HTSs. Thus, the optimized distance (*d*) between adjacent layers is 3.278 Å. The calculated binding energy is found to be −71 meV, suggesting a typical vdW HTS. In Fig. 6(c), we present the corresponding band structure for SnC_2_Br film. Bader analysis^43^ indicates no charge transfer between adjacent layers, thus the states near the Fermi level are mainly determined by SnC_2_Br film. In comparison to the free-standing film, little difference is observed between them. As a result, it is indeed a robust QSH insulator with band inversion not being affected by the *h*-BN substrate. Similar electronic properties of SnC_2_X@BN (X = H, F, Cl, I) HTSs are obtained, as shown in Fig. S6.

To conclude, on the basis of first-principles calculations, we predict a class of new QSH insulator of SnC_2_X films with sizable bulk gaps, making the experimental observation facile. Noticeably, the SnC_2_Cl, SnC_2_Br, and SnC_2_I are all QSH insulators with global gaps of ~0.2 eV, while the SnC_2_H and SnC_2_F can be transformed into QSH insulator under the tensile strains. This large-gap opening is mainly attributed to the result of the strong SOC related to the *p*_x_ and *p*_y_ orbitals of Sn atoms at Γ point, significantly different from that of the *p*_z_ orbital as in pristine group IV ones. A single pair of topologically protected helical edge states are established for the edge of these systems with the Dirac point locating at the bulk band gap, and their QSH states are also confirmed with Z_2_ = 1. Also, these films on h-BN substrate are observed to support a nontrivial large-gap QSH, which harbors a Dirac cone lying within the band gap. These findings may shed new light in future design and fabrication of large-gap QSH insulators based on 2D honeycomb lattice in spintronics.

## Methods

To study the structural and electronic properties of SnC_2_X films, we employed state-of-the-art *ab initio* simulations, based on density functional theory (DFT) as implemented in VASP^44^,^45^. We used the generalized gradient approximation for the exchange and correlation potential, as proposed by Perdew-Burk-Ernzerhof (PBE)^46^, the projector augmented wave potential (PAW)^47^ to treat the ion-electron interactions. The energy cutoff of the plane waves was set to 500 eV with the energy precision of 10^−6^ eV. The Brillouin zone was sampled by using a 21 × 21 × 1 Gamma-centered Monkhorst-Pack grid. The vacuum space was set to 20 Å to minimize artificial interactions between neighboring slabs. SOC was included by a second vibrational procedure on a fully self-consistent basis. With the optimized structures, the more sophisticated HSE06 hybrid functional^37^,^38^ was used to check the corresponding results of the systems. The phonon spectra were calculated using a supercell approach within the PHONON code^48^. To determine the Z_2_ index of the inversion symmetric 2D materials, the invariants *v* can be derived from the parities of wave function at the four TRIM points K_i_, namely one Γ point and three equivalent M points in the Brillouin zone. The topological invariant ν was determined based on the Fu-Kane’s formula^49^:


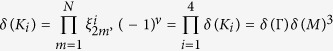


where *δ* is the product of parity eigenvalues at the TRIM points, *ξ* =  ±1 are the parity eigenvalues and *N* is the number of the occupied bands. According to the Z_2_ classification, *ν* = 1 characterizes a QSH insulator, whereas *ν* = 0 represents a trivial band topology.

## Additional Information

**How to cite this article**: Zhang, R.-w. *et al.* Room Temperature Quantum Spin Hall Insulator in Ethynyl- Derivative Functionalized Stanene Films. *Sci. Rep.* **6**, 18879; doi: 10.1038/srep18879 (2016).

## Supplementary Material

Supplementary Information

## Figures and Tables

**Table 1:** The lattice constant *a*, Sn-Sn bond length *d*_1_, Sn-X bond length *d*_2_, buckling parameters *h*, bulk band gap with SOC *E*_g_, and phase of the structures investigated in our study.

System	*a*(Å)	*d*_1_(Å)	*d*_2_(Å)	*h*(Å)	*E*_*g*_ (meV)	Phase
Stanene	4.674	2.830	–	0.852	93	TI
SnC_2_H	4.756	2.851	2.108	0.766	28	NI
SnC_2_F	4.734	2.843	2.102	0.782	25	NI
SnC_2_Cl	4.871	2.891	2.109	0.718	192	TI
SnC_2_Br	4.781	2.863	2.109	0.725	202	TI
SnC_2_I	4.811	2.874	2.112	0.709	234	TI

The phase indicates whether the material is a normal insulator (NI) or TI based on the parity calculations.

**Table 2:** Parities of occupied spin-degenerate bands at the TRIM Points for SnC_2_H and SnC_2_Br.

*Γ*_*i*_	Parity of *ξ*_2n_ of occupied bands	*δ*_*i*_	*Γ*_*i*_	Parity of *ξ*_2n_ of occupied bands	*δ*_*i*_
(0.0, 0.0)	**+−+−+−+++− − −+**	**+**	(0.0, 0.0)	**− − − −+−+− − − − −+++− − −+**	**−**
(0.5, 0.0)	**+−+−+− −+++− −+**	**+**	(0.5, 0.0)	**− − − −+−+− − − − −+++− − −+**	**−**
**(0.0, 0.5)**	**+−+−+− −+++− −+**	**+**	(0.0, 0.5)	**− − − −+−+− − − − −+++− − −+**	**−**
(0.5, 0.5)	**−+−+−++− − −++−**	**−**	(0.5, 0.5)	**− − − − −+−+− − − −+− − −++−**	**+**
*ε* ≥ 2.0% SnC_2_H	**Ζ**_**2**_ **topological invariant**	**ν** = **1**	***ε* ≥ −2.0% SnC_2_Br**	**Ζ**_**2**_ **topological invariant**	**ν** = **1**

Here, we display the parities of 13 occupied spin-degenerate bands for SnC_2_H (*ε* ≥ 2.0%) and the parities of 19 occupied spin-degenerate bands for SnC_2_Br (*ε* ≥ −2.0%). Positive and negative signs denote even and odd parities, respectively. The sign in parentheses is the product of the parity eigenvalues of the occupied spin-degenerate bands.
