# Supplementary material for: Room Temperature Quantum Spin Hall Insulator in Ethynyl-Derivative Functionalized Stanene Films
Source: Sci Rep. 2016 Jan 5;6:18879. doi: 10.1038/srep18879 (PMC4700436; doi:10.1038/srep18879)
Supplement: Supplementary Information [file srep18879-s1.doc]

**Supplementary Information for:**

**Room Temperature Quantum Spin Hall Insulator in Ethynyl- Derivative Functionalized Stanene Films**

Run-wu Zhang,^a^ Chang-wen Zhang*,^a^ Wei-xiao Ji,^a^ Shen-shi Li,^b,a^ Shi-shen Yan,^b^ Shu-jun Hu,^b^ Ping Li,^a^ Pei-ji Wang ^a^ and Feng Li^a^

^a^School of Physics and Technology, University of Jinan, Jinan, Shandong, 250022, People’s Republic of China

^b^School of Physics, State Key laboratory of Crystal Materials, Shandong University, Jinan, Shandong, 250100, People’s Republic of China

* Corresponding author: C. W. Zhang: [zhchwsd@163.com](mailto:zhchwsd@163.com)


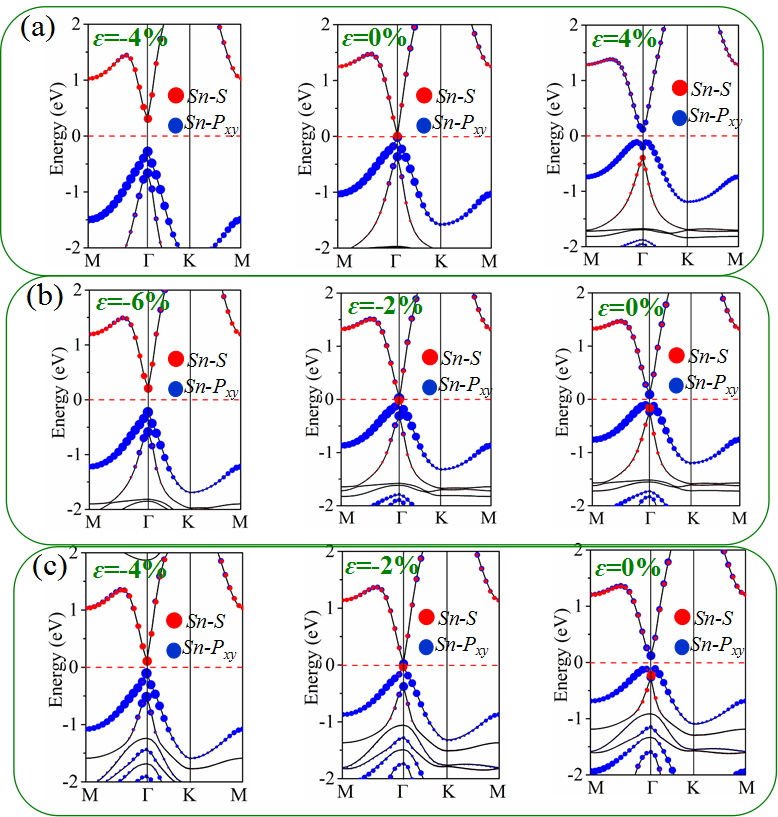


**Fig. S1** Orbital-resolved band structures with SOC for (a) SnC_2_F, (b) SnC_2_Cl, and (d) SnC_2_I, respectively. The red dots represent the contributions from the *s* orbital of Sn atoms, while the blue dots represent contributions from the *p*_x_ and *p*_y_ atomic orbitals of Sn atoms.


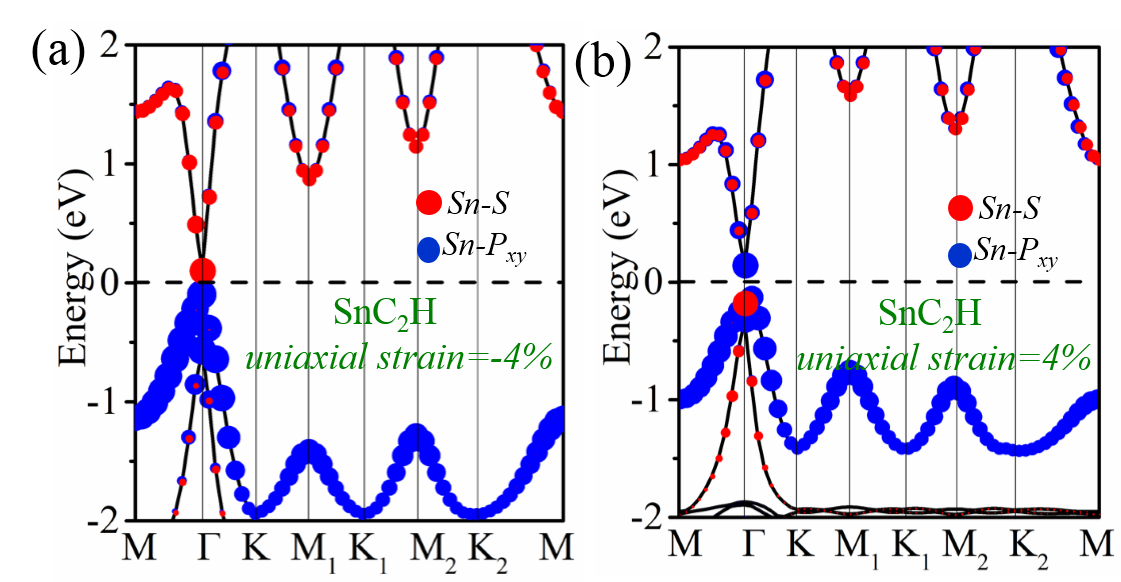


**Fig. S2** Orbital-resolved band structures of SnC_2_H under uniaxial strain (ε = -4% and ε = 4%).

**
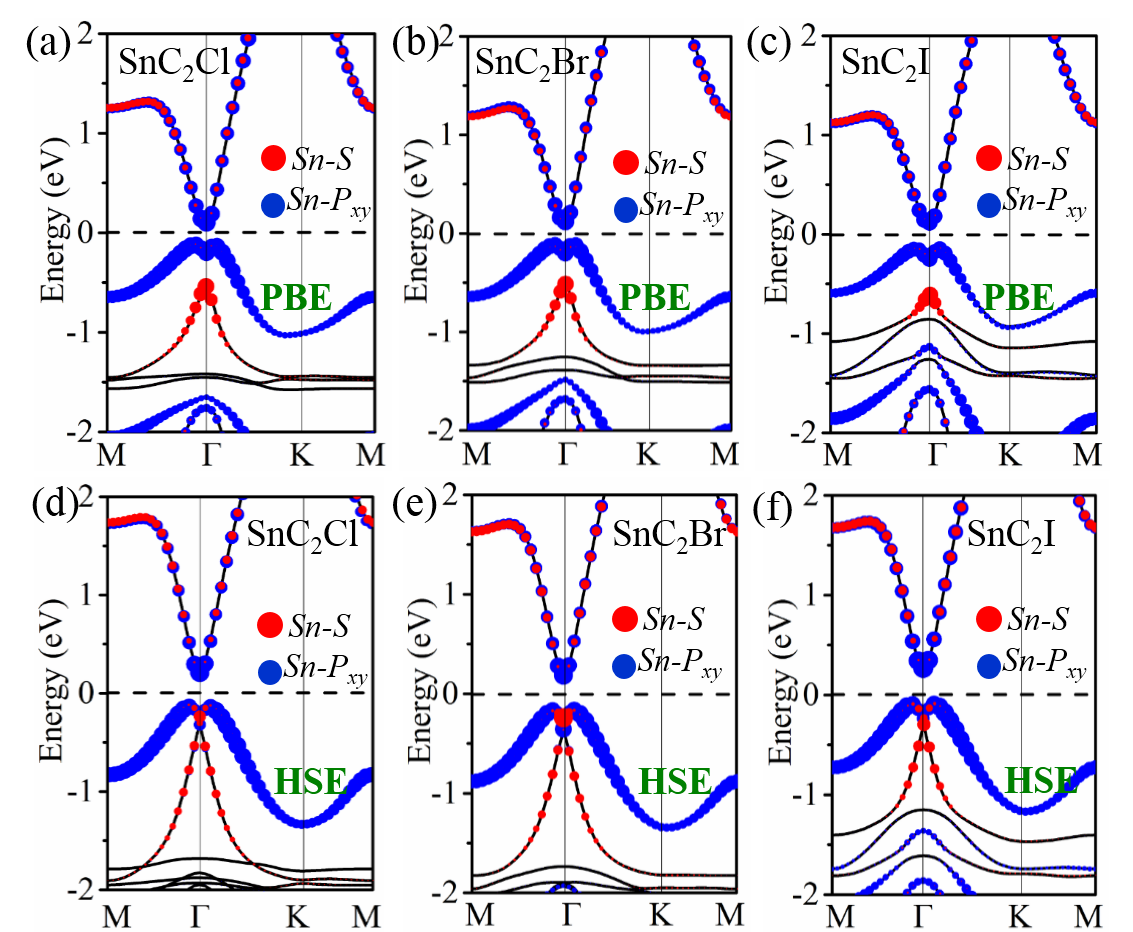
**

**Fig. S3**. The calculated band structures of (a) SnC_2_Cl (PBE), (b) SnC_2_Br (PBE), (c) SnC_2_I (PBE) and (d) SnC_2_Cl (HSE), (e) SnC_2_Br (HSE), (f) SnC_2_I (HSE), respectively.

**
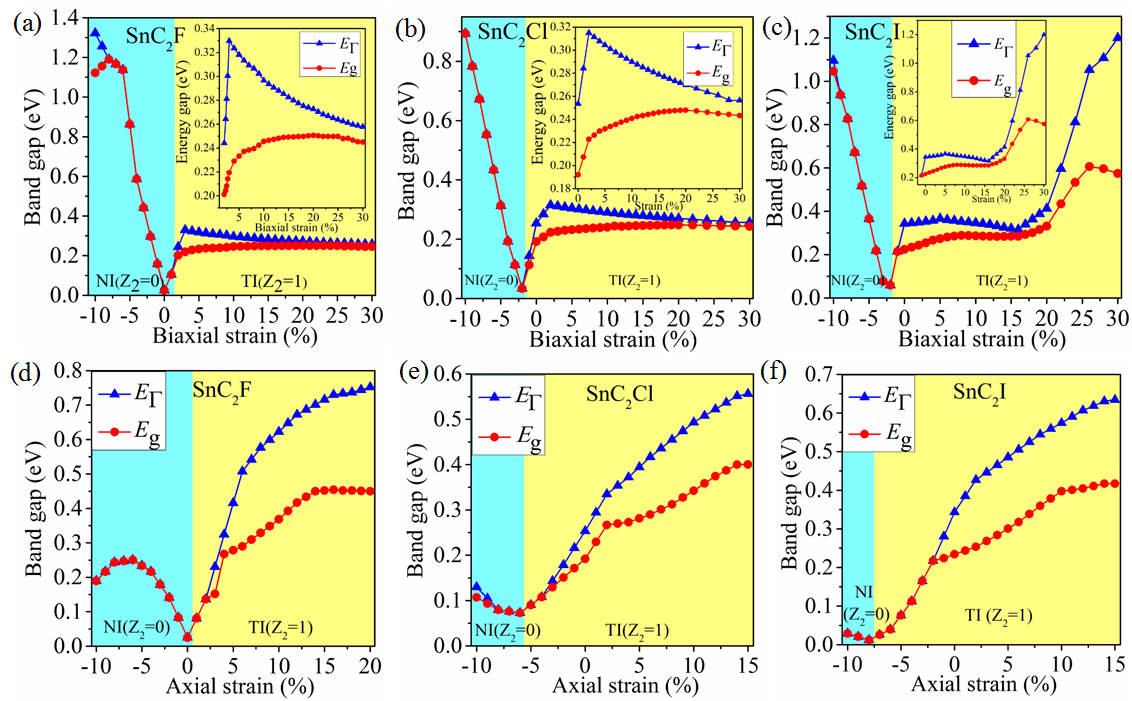
**

**Fig. S4** The calculated band gaps at Γ point (*E*_Γ_) and global gaps (*E*_g_) of SnC_2_X (X= F, Cl, I) as a function of biaxail and uniaxial strains. Notably, the insets in panel show the trend of band gaps of TI phase as a function of biaxial strain.

**
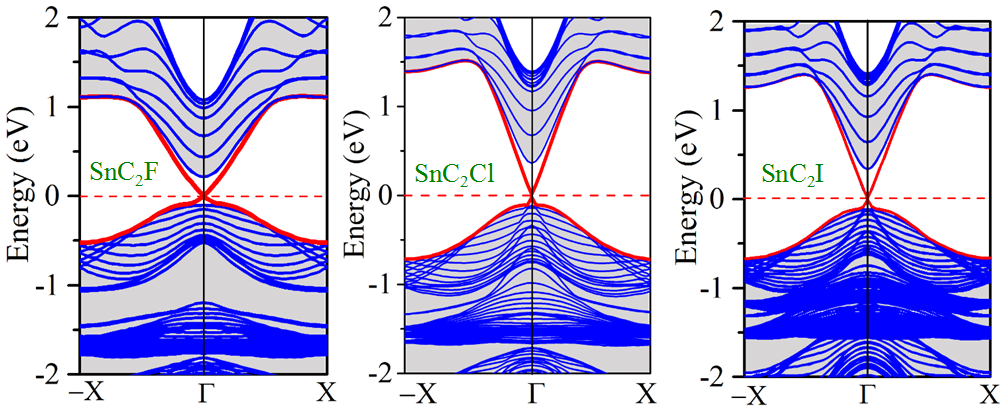
**

**Fig. S5** Calculated electronic band structures of a zigzag-type-edged nanoribbon from SnC_2_X (X = F, Cl, I). The helical edge states are indicated by the red lines.

**
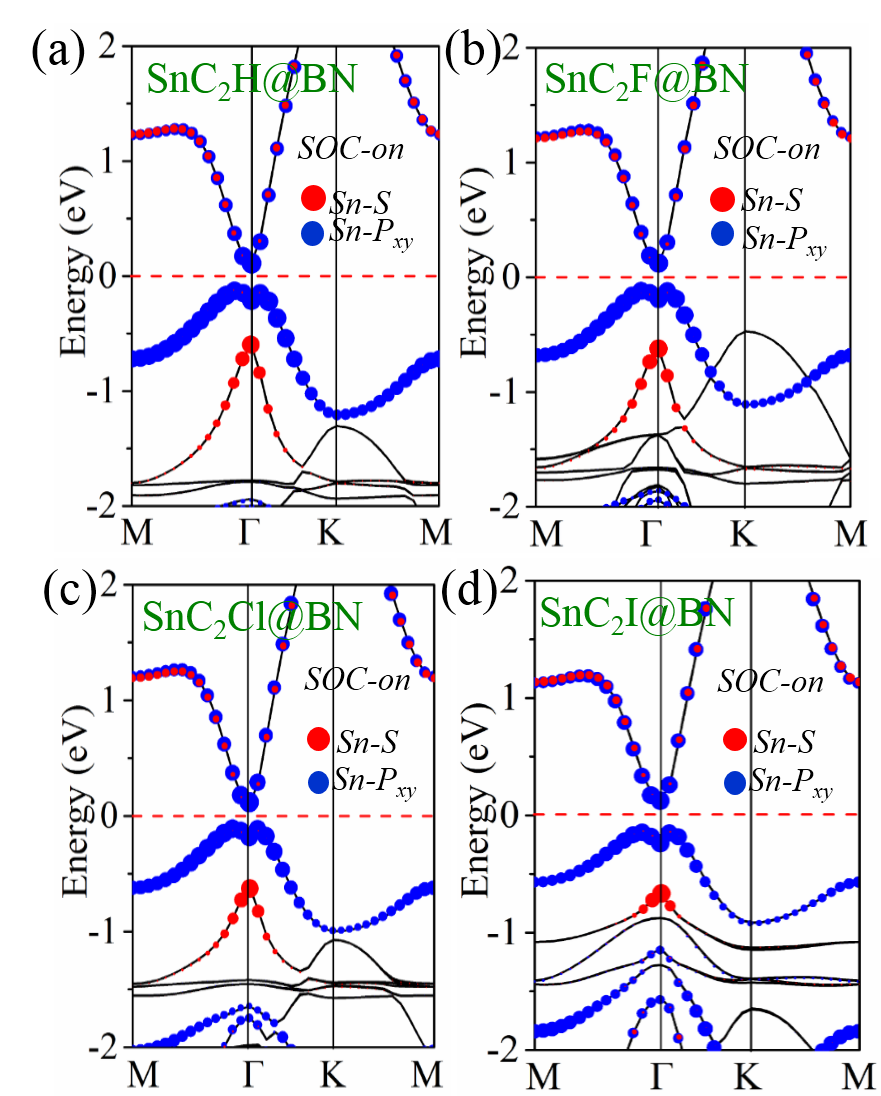
**

**Fig. S6** Orbital-resolved band structures of SnC_2_X (H, F, Cl, I)@BN HTSs with SOC.
